# P03 Our pragmatic approach to the monitoring of blood parameters during oral antibiotic therapy for complex infection

**DOI:** 10.1093/jacamr/dlaf239.007

**Published:** 2026-01-05

**Authors:** Shanya Sivakumaran, Charlotte Richards, Thomas Morris

**Affiliations:** Public Health Wales, UK; Swansea Bay University Health Board, Swansea, UK; Swansea Bay University Health Board, Swansea, UK; Public Health Wales, UK; Swansea Bay University Health Board, Swansea, UK

## Abstract

**Background:**

The use of oral antibiotics for the treatment of complex infections has been increasingly adopted in our local practice due to accumulating evidence of comparable outcomes with IV therapy. However, the optimum monitoring of blood parameters for these patients in routine practice is unclear, especially where the adverse events being screened for (hepatotoxicity, nephrotoxicity, cytopenia) are rare. Though we have local recommendations for monitoring of oral agents, with no formal complex outpatient oral antibiotic therapy (COpAT) service locally, the responsibility of the monitoring is generally left to individual clinical specialties. In the last year, feedback was received from orthopaedic colleagues, one of our main prescribers of COpAT, that complying with our recommendations for monitoring was difficult, due to issues including limitations around local outpatient phlebotomy capacity, provision of a tertiary service for patients out of area, and ability to follow up outpatient results in a timely manner. A brief appraisal of our guidance raised concern that the importance of monitoring of higher toxicity antimicrobials (such as linezolid, co-trimoxazole) could be lost in a sea of suggestions for lower risk antimicrobials, and that whilst we wanted to ensure monitoring was conducted where warranted, this had to be balanced with the potential for unnecessary testing, which has consequences for both the individual patient and the healthcare system.

**Objectives and methods:**

We therefore undertook a full review of our guidance, which involved consulting infection colleagues in other centres to learn more about practice around the country, discussion with key stakeholders locally, and examination of relevant literature. The latter included formal recommendations in the Summary of Product Characteristic and British National Formulary (BNF) for individual drugs, primary data and review papers, and infection society guidelines.

**Results:**

Overall, we felt there was a paucity of evidence to inform guidance, largely because population level studies to date often lack the ‘denominator data’ needed for estimating prevalence. In our updated guidance, we therefore now take a BNF-based approach regarding which antimicrobials need monitoring. This led to removal of our previous recommendations for the routine monitoring of amoxicillin, flucloxacillin, doxycycline and fluoroquinolones. For those agents where monitoring is suggested by the BNF, in the absence of national or international recommendations regarding the frequency of monitoring, these were agreed after local discussion and consensus. For co-trimoxazole, this involved liaising with renal colleagues, and we now take a risk-based approach to suggested frequencies and provide advice regarding risk factors for hyperkalaemia and interpretation of creatinine rises.

**Conclusions:**

In summary, review of our local guidance has removed the requirement for routine monitoring for selected low risk antimicrobials, and conversely, provides more detailed recommendations around monitoring for agents associated with higher toxicity. This aims to ensure efforts are focused where they are most likely to have a patient safety benefit. Though we have taken a pragmatic approach due to the limited evidence available, we feel this area deserves further attention in order to help local teams balance patient safety and appropriate resource utilization. Capturing data which could inform a national clinical consensus is likely key to this.

